# CLING: Candidate Cancer-Related lncRNA Prioritization via Integrating Multiple Biological Networks

**DOI:** 10.3389/fbioe.2020.00138

**Published:** 2020-03-10

**Authors:** Jizhou Zhang, Yue Gao, Peng Wang, Hui Zhi, Yan Zhang, Maoni Guo, Ming Yue, Xin Li, Dianshuang Zhou, Yanxia Wang, Weitao Shen, Junwei Wang, Jian Huang, Shangwei Ning

**Affiliations:** ^1^College of Bioinformatics Science and Technology, Harbin Medical University, Harbin, China; ^2^Department of Respiratory Medicine, The Second Affiliated Hospital of Harbin Medical University, Harbin, China; ^3^School of Life Sciences and Technology, University of Electronic Science and Technology of China, Chengdu, China

**Keywords:** lncRNA, pan-cancer, web-based server, multi-dimension data fusion, network-centric prioritization

## Abstract

Identification and characterization of lncRNAs in cancer with a view to their application in improving diagnosis and therapy remains a major challenge that requires new and innovative approaches. We have developed an integrative framework termed “CLING”, aimed to prioritize candidate cancer-related lncRNAs based on their associations with known cancer lncRNAs. CLING focuses on joint optimization and prioritization of all candidates for each cancer type by integrating lncRNA topological properties and multiple lncRNA-centric networks. Validation analyses revealed that CLING is more effective than prioritization based on a single lncRNA network. Reliable AUC (Area Under Curve) scores were obtained across 10 cancer types, ranging from 0.85 to 0.94. Several novel lncRNAs predicted in the top 10 candidates for various cancer types have been confirmed by recent biological experiments. Furthermore, using a case study on liver hepatocellular carcinoma as an example, CLING facilitated the successful identification of novel cancer lncRNAs overlooked by differential expression analyses (DEA). This time- and cost-effective computational model may provide a valuable complement to experimental studies and assist in future investigations on lncRNA involvement in the pathogenesis of cancers. We have developed a web-based server for users to rapidly implement CLING and visualize data, which is freely accessible at http://bio-bigdata.hrbmu.edu.cn/cling/. CLING has been successfully applied to predict a few potential lncRNAs from thousands of candidates for many cancer types.

## Introduction

Cancer is a group of complex diseases involving multiple levels of alterations, including genetic, epigenetic and transcriptomic aberrations. In recent years, advancements in next-generation sequencing technology have made it feasible for researchers to study the “dark matter” in the genome, leading to the discovery of a number of long non-coding RNAs (lncRNAs). LncRNAs commonly defined as transcripts longer than 200 nucleotides in length with little or no protein coding potential, which have gained widespread attention as crucial players in diverse biological processes (Mercer et al., 2009).

The past decade has witnessed a sharp increase in research on lncRNAs involved in various cancer types (Ning et al., 2016). Current knowledge on the known number of lncRNAs represents only the tip of the iceberg. One of the crucial goals in the field of cancer is to fill the gap in knowledge on the association between lncRNAs and cancer. Considering the enormous cost of determining all the latent associations between known lncRNAs and cancers through biological experiments, computational methods may provide a better alternative in identifying cancer-related lncRNAs. These techniques not only contribute to filtering high-risk lncRNAs as candidate molecules for further experimental validation but also facilitate our understanding of the potential mechanisms underlying cancer development at the lncRNA level.

Recently, several computational approaches have been developed to predict or prioritize disease-related candidate lncRNAs, the majority of which assume that lncRNAs associated with the same or related diseases tend to closely interact with each other in molecular networks. Different networks have been constructed to prioritize disease candidate lncRNAs, including lncRNA-lncRNA co-expression (Ren et al., 2015), lncRNA-gene co-expression (Liu and Zhao, 2016), lncRNA functional similarity (Sun et al., 2014) and lncRNA-mRNA competitive endogenous RNA (ceRNA) networks (Xia et al., 2014). This network-based scheme has shown significant efficacy in identification of potential lncRNA-disease associations. However, the obvious limitations are that these network-based methods use just one specific type of network, and incompleteness and even false-positive data potentially limit their predictive ability. Furthermore, despite the availability of multiple lncRNA-related networks, efficient methods to integrate these different network types are lacking.

Here, we have developed a method designated as CLING, a new cancer lncRNA prioritization technique that provides overall ranking of all candidates by integrating distinct optimization results generated from nine lncRNA-centric networks. CLING has been successfully applied to predict a few potential lncRNAs from thousands of candidates for many cancer types, some of which have been confirmed in recent biological experiments.

## Materials and Methods

### Experimentally Verified Cancer lncRNAs, Genes and miRNAs

Experimentally verified cancer lncRNAs were extracted from a previous study by our group on the Lnc2Cancer database considered as the gold standard dataset in leave-one-out cross-validation and training dataset in potential cancer–lncRNA association prediction (Ning et al., 2016). We selected 10 cancer types from TCGA, each including at least 15 relevant lncRNAs in the gold standard dataset (Supplementary Table S1). Cancer-associated genes were derived from the National Cancer Institute^1^ and DisGeNET (Pinero et al., 2015). We additionally collected miRNAs relevant in cancer from HMDD v2.0 (Li Y. et al., 2014), miR2disease (Jiang et al., 2009) and miRCancer (Xie et al., 2013).

### Data Used for Network Construction

#### LncRNA and miRNA Annotation and Sequence Data

To construct a comprehensive lncRNA data set for further analyses, we relied on the non-coding classification of GENCODE (Harrow et al., 2012), and obtained lncRNA transcript sequences. Human mature miRNA sequences were derived from the miRBase (Kozomara and Griffiths-Jones, 2014).

#### Gene Ontology Annotation Data

The GO (Ashburner et al., 2000) database provides comprehensive information describing the activities of gene products. We downloaded biological process (BP) sub-ontology of human gene for follow-up study.

#### Normal and Cancer Expression Profiles

The miRNA (Illumina HiSeq miRNASeq) and mRNA expression (Illumina HiSeq RNASeqV2) profiles of the 10 human cancers were downloaded from TCGA (as of October 2015). We subsequently obtained corresponding lncRNA expression data from the TANRIC database (Li et al., 2015).

#### Experimentally Validated lncRNA and miRNA, Protein, TF Interactions

Experimental associations between miRNAs and lncRNAs were identified in starBase v2.0 (Li J.H. et al., 2014) and DIANA-LncBase (Paraskevopoulou et al., 2013). We additionally curated lncRNA–protein interactions from starBase v2.0 and NPInter v2.0 (Yuan et al., 2014) supported by AGO CLIP-seq data. After combining data sets, 53,266 validated non-redundant human lncRNA–protein pairs were assembled, comprising 10,355 lncRNAs and 565 proteins. Furthermore, 68,676 experimentally supported lncRNA and TF associations were derived from ChIPBase (Yang et al., 2013), including 4,937 lncRNAs and 119 TFs.

#### LncRNA–mRNA ceRNA Data

We collected lncRNA–mRNA ceRNA data from LncACTdb (Wang et al., 2015), comprising 5002 pairwise associations among 329 lncRNAs and 1269 mRNAs.

### Identification of AGO-CLIP Data-Supported lncRNA–miRNA Interactions

Candidate miRNA–lncRNA interactions were predicted by two of the most commonly used and efficient computational methods with default parameters, miRanda (Miranda et al., 2006) and TargetScan (Lewis et al., 2005). Additionally, 36 human AGO-CLIP-seq datasets were collected from starBase v2.0 and integrated into the pipeline to filter the union of the predictions using the two methods (Li J.H. et al., 2014). Only the miRNA binding sites on lncRNA sequences that fully overlapped with any AGO CLIP cluster were regarded as CLIP-supported sites and the corresponding lncRNA–miRNA interactions retained for further analyses. After merging with experimentally verified associations, 109,542 validated non-redundant human lncRNA–miRNA pairs were retained, including 1634 lncRNAs and 1732 miRNAs.

### lncRNA-Centric Network Construction

#### lncRNA-lncRNA and lncRNA-mRNA Co-expression Networks

To identify the lncRNA co-expression and lncRNA–mRNA co-expression networks in each cancer type, the pearson correlation coefficient (PCC) of all lncRNA–lncRNA and lncRNA–mRNA pairs were calculated based on cancer lncRNA and corresponding mRNA expression profiles. Subsequently, sets of significantly co-expressed lncRNA–lncRNA pairs and lncRNA–mRNA pairs were screened out to constitute the two network subtypes, respectively (PCC > 0.8 and FDR < 0.01).

#### lncRNA Functional Similarity Network

Furthermore, with the advantages of BP sub-ontology, each lncRNA was functionally annotated with specific BP terms among the set of co-expressed mRNAs obtained in the lncRNA–mRNA co-expression network. Fisher’s Exact Test was performed to measure pairwise lncRNA function similarity through assessing whether the two lncRNAs significantly enriched the interacting BP terms. The test calculates the *P*-value using the following equation:

(1) $$p=\,\frac{\sum_{i=k}^{\min\left(L_{a},L_{b}\right)}\left(\begin{array}{c} L_{b}\\ k \end{array}\right)\,\left(\begin{array}{c} M-L_{b}\\ k-i \end{array}\right)}{\left(\begin{array}{c} M\\ L_{a} \end{array}\right)}$$

where *M* is the number of all BP terms in GO, and *L*_*a*_ and *L*_*b*_ are the BP terms annotated in lncRNA A and lncRNA B, respectively, and *k* represents the number of BP terms that are significantly enriched with both lncRNA A and lncRNA B. Therefore, substantial numbers of lncRNA pairs with significant functional similarities were obtained to form the lncRNA function similarity network (FDR < 0.01).

#### lncRNA-lncRNA and lncRNA-mRNA ceRNA Networks

Similarly, to build the lncRNA–lncRNA ceRNA network, a hypergeometric test was used to evaluate whether the two lncRNAs have a potential ceRNA relationship by considering their shared interactive miRNAs. As a result, we obtained a complex lncRNA–lncRNA ceRNA network composed of 186,306 associations among 1633 lncRNAs. lncRNA-mRNA ceRNA network was constructed by above data from LncACTdb.

#### lncRNA Sequence Similarity Network

Based on lncRNA transcript sequences, sequence similarities of all lncRNA pairs were predicted using BLAST+ (version 2.2) with default parameters. After rigorous filtration, 28,622 pairwise sequence similarities among 5231 lncRNAs were retained and used to construct a lncRNA sequence similarity network (*e*-value < 10^–5^ and bit score >80.0).

#### lncRNA–Protein, lncRNA–TF, and lncRNA–miRNA Interactions Networks

lncRNA–protein, lncRNA–TF, and lncRNA–miRNA interactions were constructed by transforming the corresponding interacted data obtained earlier.

### Random Walk With Restart Algorithm

Random walk with restart was performed on each lncRNA-centric network (Kohler et al., 2008). This technique can be used to prioritize potential cancer lncRNAs by simulating a random walker, starting with a set of source nodes and randomly moving to its network neighbors. Formally, RWR is defined as:

(2) $$p_{q+1}=\left(1-\alpha \right)\,W\,p_{q}+\alpha \,p_{0}$$

where *p*_0_ is the original probability vector, which is the probability of being at a source node (equal to 1 here), *W* is the column-normalized adjacency matrix of an individual network involved in CLING, αis the restart probability of the random walk at every step at the source nodes, *p*_*q*_ is a vector in which the *i*th element has the probability of being at node *i* during the time step *q*.

### Data Integration in CLING

In our method, individual prioritization results generated from each network and lncRNA topological properties are fused into an overall optimization list, which can be divided into four sections.

First, for a specific cancer *c*, similar to Endeavor, we obtain a rank ratio for the investigated candidate lncRNA in each network. Given the absence of some lncRNAs in some networks, rank ratio is defined as

(3) $$r_{i\,j}=\left\{ \begin{array}{c} \frac{R_{i\,j}}{n_{j}}\left(i\in j\right)\\ 1\left(i\notin j\right) \end{array}\right.$$

where *R*_*ij*_ is the ranking of candidate lncRNA *i* in the network *j* and *n*_*j*_ is the number of lncRNAs included in network *j*.

Second, since known cancer lncRNA betweenness and degree are significantly larger than candidates in many networks, the average normalized betweenness and degree of individual lncRNA have also been taken into consideration which can be calculated using following three substeps:

Substep 1: We normalize betweenness of candidate lncRNAs depending on whether they are missing in the corresponding network.

(4) $$b_{i\,j}=\left\{ \begin{array}{c} \frac{B_{i\,j}-B_{j\,m\,i\,n}}{B_{j\,m\,a\,x}-B_{j\,m\,i\,n}}\left(i\in j\right)\\ 0\;\left(i\notin j\right) \end{array}\right.$$

where *B*_*ij*_ is the betweenness of lncRNA *i* in network *j*, and *B*_*jmin*_ and *B*_*jmax*_ are the minimal and maximal betweenness of network *j*, respectively.

Substep 2: The average normalized betweenness of the candidate lncRNA is obtained using the equation:

(5) $$b\,s_{i}=\frac{1}{T_{i}}\,\sum_{j=1}^{N}b_{i\,j}$$

where, *T*_*i*_ is the number of the networks that the candidate lncRNA *i* is involved in.

Similarly, the average normalized degree of an individual lncRNA is acquired by:

(6) $$d_{i\,j}=\left\{ \begin{array}{c} \frac{D_{i\,j}-D_{j\,m\,i\,n}}{D_{j\,m\,a\,x}-D_{j\,m\,i\,n}}\left(i\in j\right)\\ 0\;\left(i\notin j\right) \end{array}\right.$$

(7) $$d\,s_{i}=\frac{1}{T_{i}}\,\sum_{j=1}^{N}d_{i\,j}$$

where *D*_*ij*_ is the degree of lncRNA *i* in network *j*, and *D*_*jmin*_ and *D*_*jmax*_ are the minimal and maximal betweenness of network *j*, respectively.

Third, the number of networks that the candidate lncRNA is involved in is also considered:

(8) $$t_{i}=\frac{T_{i}}{N}$$

where *N* is the number of networks used in this work.

Finally, all separate values of candidate lncRNA are combined into one overall score.

(9) $$S_{i}=\frac{1}{t_{i}*e^{b\,s_{i}+d\,s_{i}}}\,\prod_{j=1}^{N}\log\left(r_{i\,j}+1\right)$$

This overall score measures the potential relationship between lncRNA *i* and cancer *c* among whole networks. The score is subsequently used to rank all the candidate lncRNAs for a specific cancer type.

## Results

### Global Properties of lncRNA-Centric Networks

**FIGURE 1:**
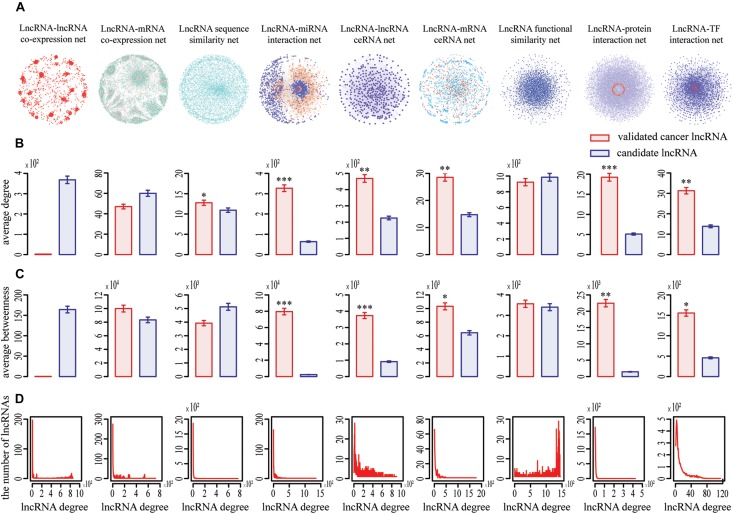
Topological properties of nine lncRNA-centric networks involved in COAD. **(A)** Nine networks used to prioritize potential COAD-related lncRNAs. **(B,C)** Known cancer lncRNAs generally had significantly higher degree and betweenness centrality than other candidates in most networks based on Wilcox rank sum test (*, **, *** representing *P* < 0.05, *P* < 0.01, *P* < 0.001, respectively). Data were presented as means ± SEM. Known cancer lncRNAs and candidates are indicated in red and blue along the *X* axis, respectively. Average degrees of these two groups of lncRNAs are specified in the *Y* axis. **(D)** All networks displayed a power law distribution, except LFS (lncRNA function similarity network). The *X* axis indicates lncRNA degree distribution, *Y* axis indicates the number of lncRNAs according to the *X* axis.

Nine complex networks were generated (Figure 1A), including lncRNA–lncRNA co-expression (LCN), lncRNA ceRNA (LCE), lncRNA function similarity (LFS), lncRNA sequence similarity (LSS), lncRNA-mRNA co-expression (LMCN), lncRNA-mRNA ceRNA (LMCE), lncRNA-miRNA interaction (LMI), lncRNA-protein interaction (LPI), and lncRNA–transcription factor (TF) interaction (LTFI) networks. Based on diverse networks, the number of lncRNAs ranging from 329 to 4937 and the number of interactions ranging from 2068 to 2,511,269 in different types of cancer, respectively (Supplementary Table S1). Among these networks, LCN, LMCN, and LFS were cancer type-specific, sourced from corresponding expression data.

We focused on the known cancer lncRNAs topological properties of each network, which revealed generally higher degree and betweenness. For example, the average degree and betweenness of validated COAD-related lncRNAs were significantly higher than those of the remaining candidates in six and five out of nine networks, respectively (Wilcoxon rank-sum test, *P* < 0.05) (Figures 1B,C), indicating that cancer lncRNAs tend to be more important within the broader context of the whole network and have synergistic communication. In addition, our data showed that eight of nine networks (except LFS) exhibited a scale-free, small-world and modular architecture, with the degree following a power law distribution (Figure 1D). Similar phenomena were detected for the nine other cancers (Supplementary Figures S1–S9).

### Performance of CLING

**FIGURE 2:**
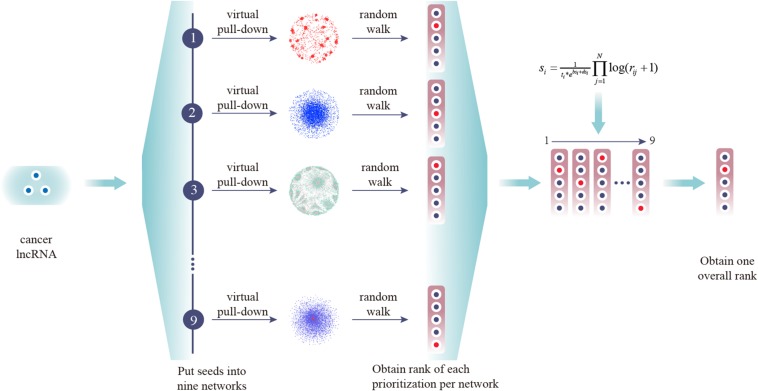
Experimental scheme of CLING. First, experimentally supported cancer lncRNAs were extracted from Lnc2cancer and virtually pulled down into nine lncRNA-centric networks. Next, all candidate lncRNAs were ranked in one prioritized list for each network based on the RWR algorithm. Finally, all separate prioritization results and lncRNA topological properties were integrated into an overall rank.

**FIGURE 3:**
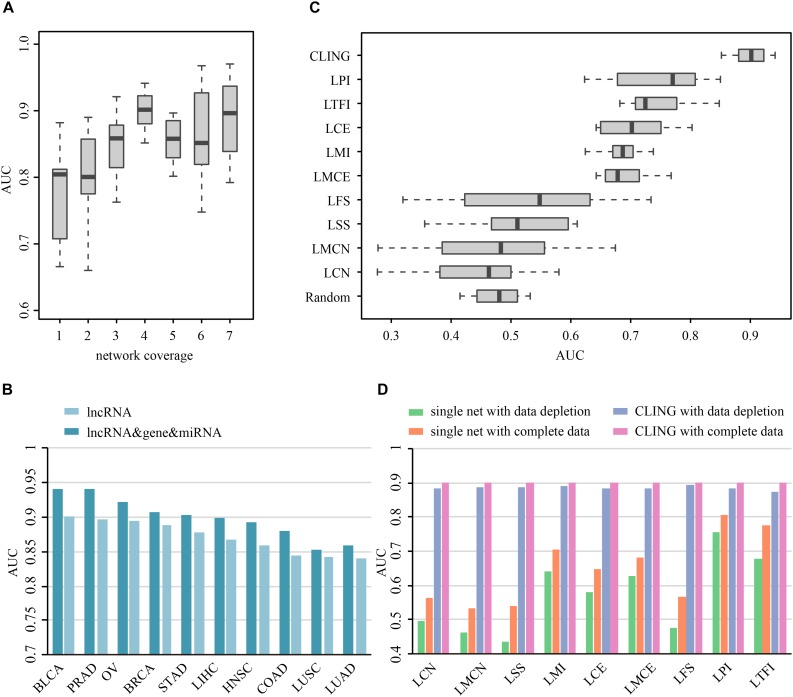
Prioritization power of CLING. **(A)** Performance evaluation of CLING based on lncRNAs with different network coverages. **(B)** Comparison of the predictive power of CLING for 10 types of cancer with consideration of other experimental cancer molecules (gene, miRNA) as additional source nodes in cancer lncRNA prioritization. **(C)** Distribution of AUC values across 10 cancer types using CLING and individual networks for cancer lncRNA annotated in each of network. **(D)** Robustness of CLING against the removal of 20% randomly selected lncRNAs as well as relevant associations in each network. Plots show the AUC scores calculated for the prediction of cancer-lncRNA associations for liver hepatocellular carcinoma (LIHC) using CLING with complete data (gray), data depletion in one network (pink), individual networks with complete data (orange), and individual networks with data depletion (green).

Prioritization of lncRNAs by CLING involved three main steps (Figure 2). As the number of known cancer lncRNAs contained in at least four out of the nine networks covered the majority of the total quantity, LOOCV and ROC analyses were further applied to investigate how the predictive power of CLING changes when prioritized lncRNAs at different network coverage. The performance of CLING was remarkably enhanced when only identifying lncRNAs involved in at least four networks, compared with consideration of all candidates (Figure 3A). Human genes (mRNA, protein) and miRNAs participating in some networks were also included to estimate whether the performance of CLING is improved with the addition of known cancer genes and miRNAs. The AUC scores across 10 cancer types revealed slight enhancement of the proficiency of CLING including this modification (Figure 3B). To demonstrate the superiority of CLING in using multiple networks to predict cancer lncRNAs, we used the distribution of AUC scores across 10 cancer types to summarize the general prediction power of CLING and individual networks (Figure 3C). CLING significantly outperformed all individual network prioritizations for the 10 cancer types in terms of AUC score. Moreover, CLING could make up for the deficiencies of individual network optimization. For instance, AUC scores yielded using LCN and LMCN in BRCA were only 0.399 and 0.2778, respectively, which escalated to 0.9077 with CLING.

To test the robustness of CLING under these circumstances, we successively removed 20% randomly selected lncRNAs as well as data related to these nodes in each network. LOOCV and ROC analyses disclosed a stable performance of CLING even under conditions of data depletion. For example, despite data depletion from different networks, CLING exhibited high predictive power for liver hepatocellular carcinoma (LIHC) with only a few tenths percentage recession in AUC values (Figure 3D). Notably, the reduction of AUC values using discrete network prioritization was approximately 10 times the decrease observed with CLING. These results clearly illustrated that CLING can overcome situations of network incompleteness and missing data, supporting its utility as a robust method for valid lncRNA identification. We further assessed the predictive power of CLING by testing whether the top 10 lncRNA candidates for each cancer type have been biologically validated as true cancer lncRNAs. By application of literature mining, among the top 10 potential cancer lncRNAs in each cancer type, three, two, one, one, one, one and one associations of STAD, COAD, BLCA, PRAD, OV, and LIHC predicted using CLING have been confirmed (Supplementary Table S2).

### Comparison of CLING With Other Methods

**FIGURE 4:**
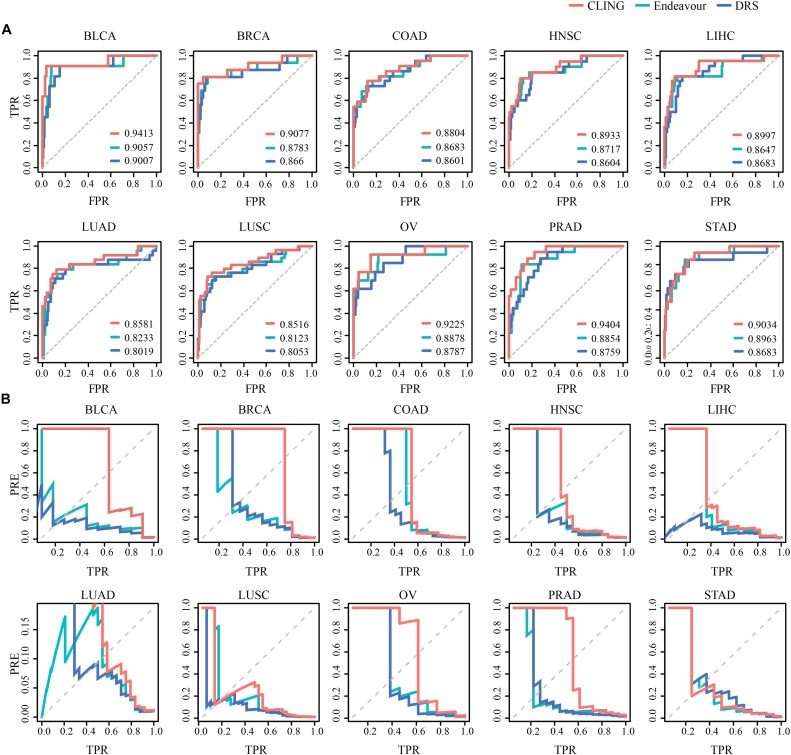
Prediction power of known cancer lncRNAs performed for CLING, Endeavor and DRS. **(A)** ROC curves indicate that CLING performed better than other methods. **(B)** Curves showing prediction precision (PRE) vs. recall (or TPR) indicate that CLING outperformed other methods.

We used two state-of-the-art methods as comparative analyses for cancer lncRNA prioritization, including Endeavor (Aerts et al., 2006) and DRS (Li and Patra, 2010). For comparative evaluation of the performance of these three methods, we replaced Endeavor and DRS with CLING and calculated the corresponding AUC values across 10 cancer types (Figure 4A). While predictive performances for three methods were comparable, CLING consistently generated the highest AUC score, followed by Endeavor and DRS, for each cancer type (Supplementary Table S3). Further, a comparison for the precision-recall (precision-TPR) curves of the CLING, DRS and Endeavor again showed that CLING is superior over other two kinds of methods, especially for high recall rate, suggesting that CLING can achieve higher accuracy and can be applied to many more cancer types (Figure 4B). The time consumed by DRS and Endeavor was dramatically higher with increased lncRNA number while CLING maintained stable efficiency over the same time period. When the number of candidate lncRNAs was as high as 10,000, CLING was >20 times faster than Endeavor. These results demonstrated that CLING achieves a more stable performance with higher efficacy in identifying cancer-related lncRNAs than the current methods through combining lncRNA properties.

### Predicted lncRNA Function Profiles in Human Cancers

**FIGURE 5:**
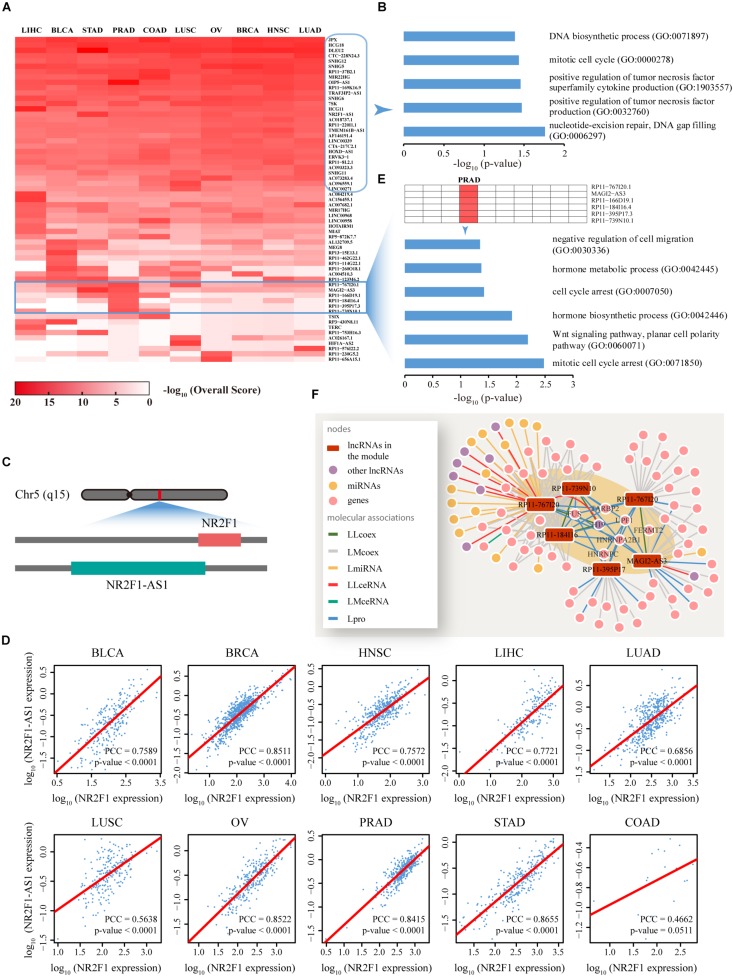
Functional analysis of the top ranked lncRNAs among 10 cancer types. **(A)** Dynamic changes in the function profiles of 62 lncRNAs (rows) in 10 types of cancer (columns). Rows and columns were ordered using two-way hierarchical clustering of the −log_10_ Overall Score between lncRNAs and cancer, whereby red/white indicates high/low −log_10_ Overall Score. In total, 29 lncRNAs displaying significant relationships with all cancer types were distributed in the upper section while the lower section illustrates isolated lncRNA modules restricted to a specific cancer type. **(B)** Cancer-related biological processes (BP) enriched for 29 lncRNAs are highly correlated with all 10 cancer types. **(C)** The lncRNA, NR2F1-AS1, is functionally involved in 10 cancers, potentially through regulation of NR2F1. A sketch diagram describes the genome locations of lncRNA NR2F1-AS1 and gene NR2F1, shown in the green and red bars, respectively. **(D)** Evaluation of the co-expression between NR2F1-AS1 and NR2F1 among 10 cancer types. For each cancer type, we plotted NR2F1-AS1 against NR2F1 expression value. High linear correlations were observed for nine cancers, with the exception of COAD. Linear fitting lines are indicated in red. *X* and *Y* axis were plotted on a log_10_ scale. **(E)** Prostate cancer-related biological processes enriched for the module composed of six lncRNAs that is functionally restricted to PRAD. **(F)** A composite subnet formed by the six lncRNAs involved in PRAD and their direct neighbors in each network showing the interactions between these molecules. The shaded area indicates an observable module of the six lncRNAs through tight connections with known prostate cancer-related lncRNAs, genes and proteins. LLcoex, lncRNA–lncRNA co-expression; LMcoex, lncRNA–mRNA co-expression; LmiRNA, lncRNA–miRNA interactions; LLceRNA, lncRNA–lncRNA ceRNA; LMceRNA, lncRNA–mRNA ceRNA; Lpro, lncRNA–protein interactions.

To predict specific lncRNA functions among human cancers, for each cancer type, the top 10 novel lncRNAs not reported to be associated with any cancer type as well as the relevant overall scores across 10 cancers were screened out (Figure 5A). Functional enrichment analysis based on Enrichr (Kuleshov et al., 2016) suggested that these lncRNAs are significantly associated with several fundamental cancer-related BP (*P* < 0.05) (Figure 5B). Specific genomic analyses facilitated the delineation of one of these lncRNAs, termed NR2F1-AS1, as a 176,293 bp gene with 14 non-protein coding transcriptional variants located on chromosome 5q15. Interestingly, a gene denoted NR2F1 that plays a critical role in the development of peripheral nervous and central nervous systems (Pereira et al., 2000) is located diagonally opposite NR2F1-AS1 in the human genome (Figure 5C). Further co-expression pattern analyses demonstrated highly consistent co-expression (*P* < 0.0001) of NR2F1-AS1 and NR2F1 across nine of the cancer types examined [with the exception of COAD due to the limited tumor sample number (18) used for calculations] (Figure 5D). We also found that NR2F1-AS1 is differential expressed in many types of cancers such as BLCA and OV (Supplementary Figure S10). Thus, based on the principle of guilt-by-association, we propose that the lncRNA NR2F1-AS1 is involved in the regulation of multiple cancers through exerting effects on NR2F1.

In addition to lncRNAs commonly participating in all 10 cancers, the profile revealed isolated lncRNA blocks or modules, which were restricted to a specific cancer type (Figure 5A). For example, a module composed of six lncRNAs involved in prostate cancer was significantly enriched with prostate cancer-related BP (*P* < 0.05) (Figure 5E). Intensive network analyses based on the subnet formed by these six lncRNAs and their direct neighbors in each network also revealed that these lncRNAs comprised a tightly connected module through direct associations with experimentally validated prostate cancer-related lncRNAs or proteins, such as H19, FUS, and TARBP2 (Figure 5F).

### Case Study

To verify the advantages of CLING in identifying cancer lncRNAs, we compared the lncRNA rank lists of Liver hepatocellular carcinoma (LIHC) acquired using CLING and DEA. The correlation coefficient between CLING Overall Score list and DEA −log_10_ (*P*-values) was 0.0759 (*P* < 0.0001). In particular, we manually assessed the expression patterns of the top 20 candidates predicted by CLING that are considered to be LIHC-related lncRNAs with high possibility (Supplementary Table S4). Notably, 10 of the lncRNAs showed significantly different expression between tumor and normal liver samples (*P* < 0.05, FC > 2.0) while the remaining 10 could not be identified by DEA (*P* < 0.05, FC > 2.0, or FC < 0.5), including NEAT1 (ranked 5th by CLING) and XIST (ranked 11th by CLING), which have been verified as LIHC-associated lncRNAs in recent literature (Guo et al., 2015; Zhuang et al., 2016). Five of the remaining eight lncRNAs have already been reported as cancer lncRNAs. HCG11, ranked 1st by CLING, has been identified as a significant prognostic marker in breast cancer (Liu et al., 2016). SNHG5 and JPX ranked 6th and 7th by CLING, are implicated in gastric cancer (Zhao et al., 2016) and breast cancer (Huang et al., 2016), respectively. OIP5-AS1is reported to reduce proliferation of cervical cancer by serving as a sponge ceRNA for HuR (Kim et al., 2016) and ranked 10th by CLING. In addition, MIR22HG, ranked 19th by CLING, is a potential prognostic biomarker for LUAD (Li et al., 2016).

**FIGURE 6:**
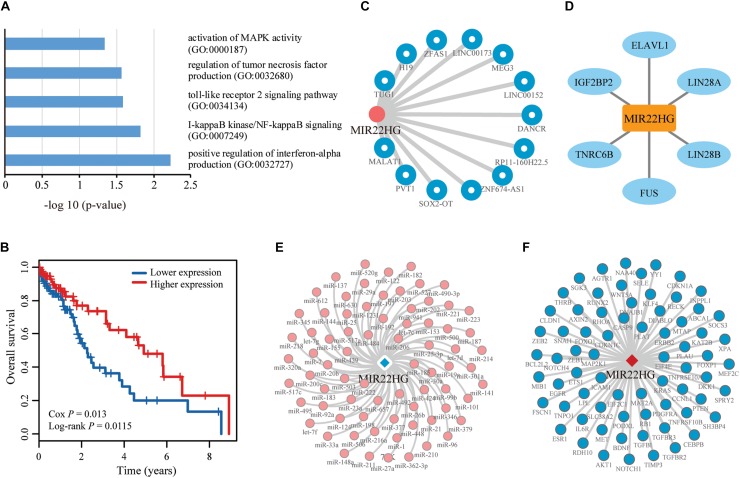
Function and network analyses of the top prioritized lncRNA in LIHC. **(A)** LIHC-related GO biological processes enriched for the top 20 lncRNAs identified using CLING. **(B)** Kaplan–Meier curves of two patient groups with higher (top 50%; *n* = 100) or lower (bottom 50%; *n* = 100) expression of MIR22HG in LIHC. Survival days are shown along the *X* axis and Overall survival rates along the *Y* axis. **(C–F)** Subgraphs formed by lncRNA MIR22HG and its directed neighbors associated with LIHC in LCE, lncRNA ceRNA network; LPI, lncRNA–protein interaction network; LMI, lncRNA–miRNA interaction network, and LMCE, lncRNA–mRNA ceRNA network, respectively.

We further performed enrichment analysis for GO BP using Enrichr. Six BP were enriched for cell cycle regulation and apoptosis, LIHC carcinogenesis and development (Figure 6A). We also performed survival analysis in LIHC patients to evaluate whether the uppermost 20 lncRNAs are potentially valuable biomarkers for predicting survival of patients. Overall, 3 of the 20 lncRNAs showed significant positive or negative relationships between expression and OS in LIHC (*P* < 0.05) (Figure 6B), suggesting a latent association with clinical outcome. Notably, one of the lncRNAs, MIR22HG, that could not be identified by DEA showed a negative correlation between expression and OS, suggestive of a tumor suppressor role in LIHC. Network analysis revealed that MIR22HG is directly associated with abundant experimentally validated LIHC-related lncRNAs, miRNAs, genes and proteins in different networks (Figures 6C–F). CLING facilitated the discovery of many LIHC candidate lncRNAs previously implicated in other cancer types but overlooked by DEA. These results support a complementarity between the two cancer lncRNA prediction methods, CLING and DEA.

### CLING Software Availability

We have developed a web-based server for users to implement CLING with a view to rapidly accessing and visualizing data pertinent to their research. The working principles and user manual of CLING can be accessed on the “HELP” page. CLING is freely accessible for non-commercial use at http://bio-bigdata.hrbmu.edu.cn/cling/.

## Discussion

The success of CLING can be attributed to a combination of several aspects. First, CLING displayed effective power in accessing several lncRNA-centric networks, which significantly differentiates it from conventional methods designed to access only one or two networks. Second, three out of nine networks used in this study (LCN, LMCE and LFS) were cancer type-specific. CLING could detect potential cancer lncRNAs for individual cancer types more efficiently. Third, CLING also makes sufficient use of the topological properties of lncRNAs implicated in each network, which further aid in the identification of true cancer-associated lncRNAs.

In the current study, due to finite lncRNA and cancer data availability, only nine networks have been introduced into CLING. The advantage of our method is integrating multiple types of biological networks which could provide more global and effective information for identifying cancer-related lncRNAs. Network-based method is an advantaged way to construct interactions between lncRNAs or other types of RNAs. One of the limitations of our method is identifying cancer-related lncRNAs for some specific cancer types are not available because of CLING was based on random walk and this method need known seed. For some special cancer types, the number of known cancer-related lncRNAs is small. More and accurate predicted cancer-related lncRNAs would be identified based on CLING as the number of validated cancer-related lncRNAs increases. More cancer types and cancer-related genes and lncRNAs would be used for predicting cancer-related lncRNAs based on updated TCGA portal in the future work. Optimization power would be improve further when new and better lncRNA and cancer datasets become available and genome annotation and curation processes are finalized. Another limitation of our study is that the purpose of this study was to develop a network-based method to predicted cancer-related lncRNAs, some of the identified lncRNAs in certain cancers should be further validated *in vitro* and *in vivo* studies.

## Conclusion

In summary, we have presented a time- and cost-effective computational method that effectively aids in the identification of cancer-relevant lncRNAs through integrative analyses of multiple networks. CLING provides additional avenues for the optimal utilization of publicly available genomic data to characterize the functions and underlying mechanisms of lncRNAs in human cancers.

## Data Availability Statement

All data included in this study are available upon request by contact with the corresponding author.

## Author Contributions

SN, JH, and JW: study design and supervision. JZ, YG, and PW: data acquisition and analysis. JZ and YG: method construction. HZ, YZ, MG, and MY: method validation. JZ, XL, DZ, YX, and WS: web server construction. All authors contributed to the manuscript and approved the final manuscript.

## Conflict of Interest

The authors declare that the research was conducted in the absence of any commercial or financial relationships that could be construed as a potential conflict of interest.

## Footnotes

1 https://www.cancer.gov

## Abbreviations

AUC: area under curve
BLCA: bladder urothelial carcinoma
BP: biological process
BRCA: invasive breast carcinoma
ceRNA: competitive endogenous RNA
CLING: cancer LncRNA identification by network grouping
COAD: colon adenocarcinoma
DEA: differential expression analyses
DRS: discounted rating system
GO: gene ontology
HNSC: head and neck squamous cell carcinoma
LCE: lncRNA ceRNA
LCN: lncRNA co-expression
LFS: lncRNA function similarity
LIHC: liver hepatocellular carcinoma
LMCE: lncRNA-mRNA ceRNA
LMCN: lncRNA-mRNA co-expression
LMI: lncRNA-miRNA interaction
lncRNA: long non-coding RNA
LPI: lncRNA-protein interaction
LSS: lncRNA sequence similarity
LTFI: lncRNA-transcription factor (TF) interaction
LUAD: lung adenocarcinoma
LUSC: lung squamous cell carcinoma
OV: ovarian serous cystadenocarcinoma
PCC: pearson correlation coefficients
PRAD: prostate adenocarcinoma
PRE: precision
ROC: receiver operating characteristic
RWR: random walk with restart
STAD: stomach adenocarcinoma
TCGA: the cancer genome atlas
TPR: true positive rate.
