# Supplementary material for: CLING: Candidate Cancer-Related lncRNA Prioritization via Integrating Multiple Biological Networks
Source: Front Bioeng Biotechnol. 2020 Mar 10;8:138. doi: 10.3389/fbioe.2020.00138 (PMC7077056; doi:10.3389/fbioe.2020.00138)
Supplement: Supplementary file 1 [file Data_Sheet_1.pdf]

CLING: Candidate cancer-related lncRNA prioritization via integrating multiple biological networks

Supplementary Data

Supplemental Figures S1-S9. Topological properties of nine lncRNA-centric networks involved in BLCA (Figure S1), BRCA (Figure S2), HNSC (Figure S3), LIHC (Figure S4), LUAD (Figure S5), LUSC (Figure S6), OV (Figure S7), PRAD (Figure S8), and STAD (Figure S9). **(A)** Nine networks used to prioritize potential cancer-related lncRNAs. **(B, C)** Known cancer lncRNAs generally showed significant higher degree and betweenness centrality than other candidates in most networks (*, **, *** signifying $P < 0.05$, $P < 0.01$, $P < 0.001$, respectively). Data are presented as means ±SEM. Known cancer lncRNAs and candidates are indicated in red and blue along the X axis. Average degrees of these two groups of lncRNAs are specified along the Y axis. **(D)** All networks displayed a power law distribution except LFS (lncRNA function similarity network). X axis indicates lncRNA degree distribution, Y axis indicates the number of lncRNAs according to the X axis.

Supplemental Figure S10. The expression level of lncRNA NR2F1-AS1 in diverse cancer typs. The red and green points represent expression of NR2F1-AS1 in tumor and normal samples. Red and green names of cancers represent up- and down-regulation.

Supplementary Table S1. Number of nodes (lncRNAs) and edges involved in the nine networks across 10 cancers.

Supplementary Table S2. Summary of literature-verified lncRNAs.

Supplementary Table S3. Time requirements of all three data integration methods.

Supplementary Table S4. CLING-ranked list of the top 20 candidate LIHC-related lncRNAs

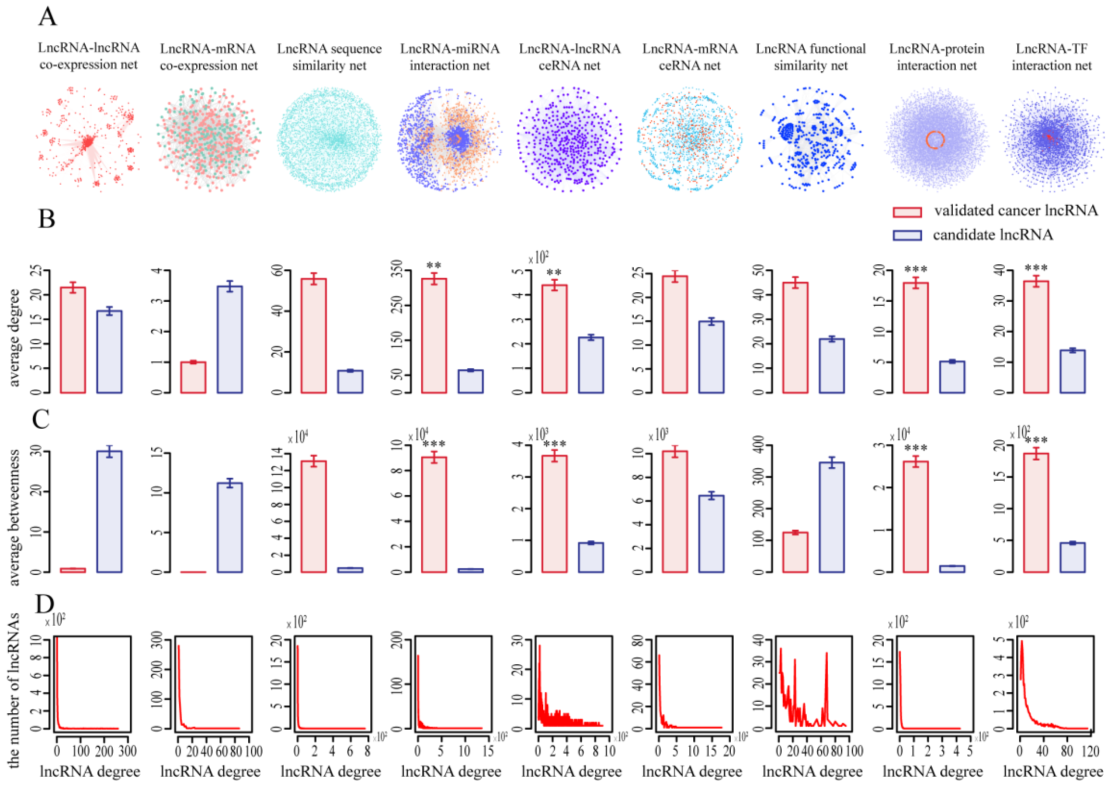

Supplementary Figure S1

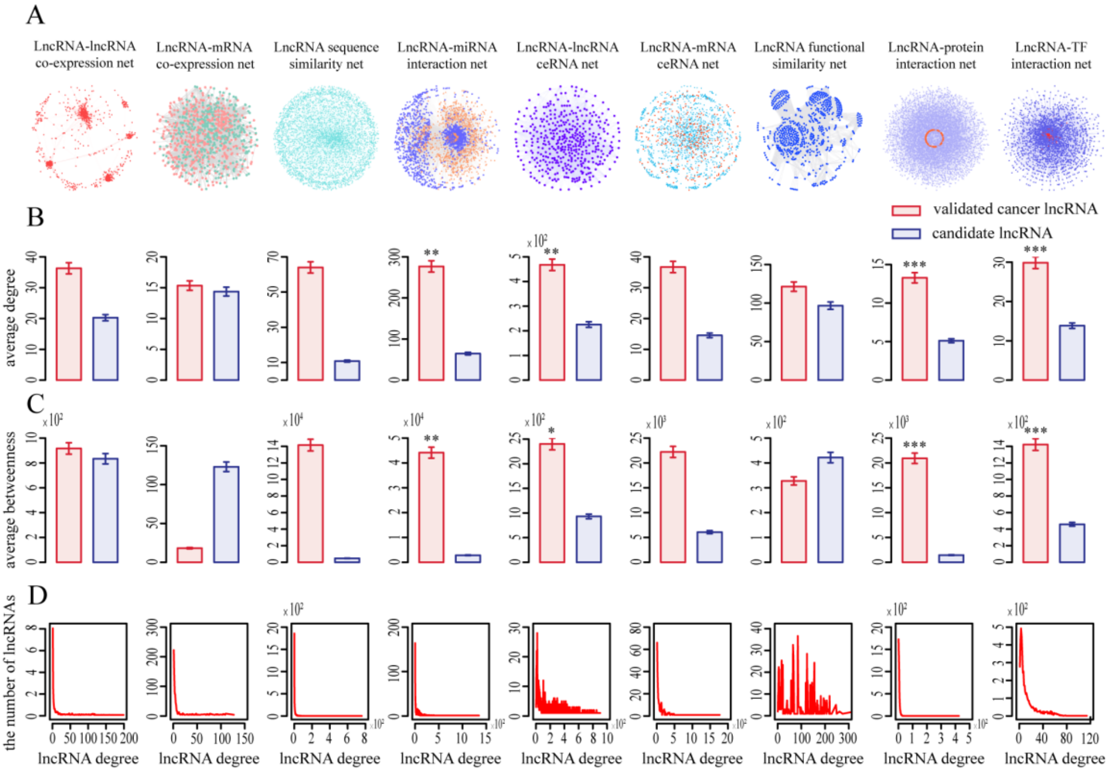

Supplementary Figure S2

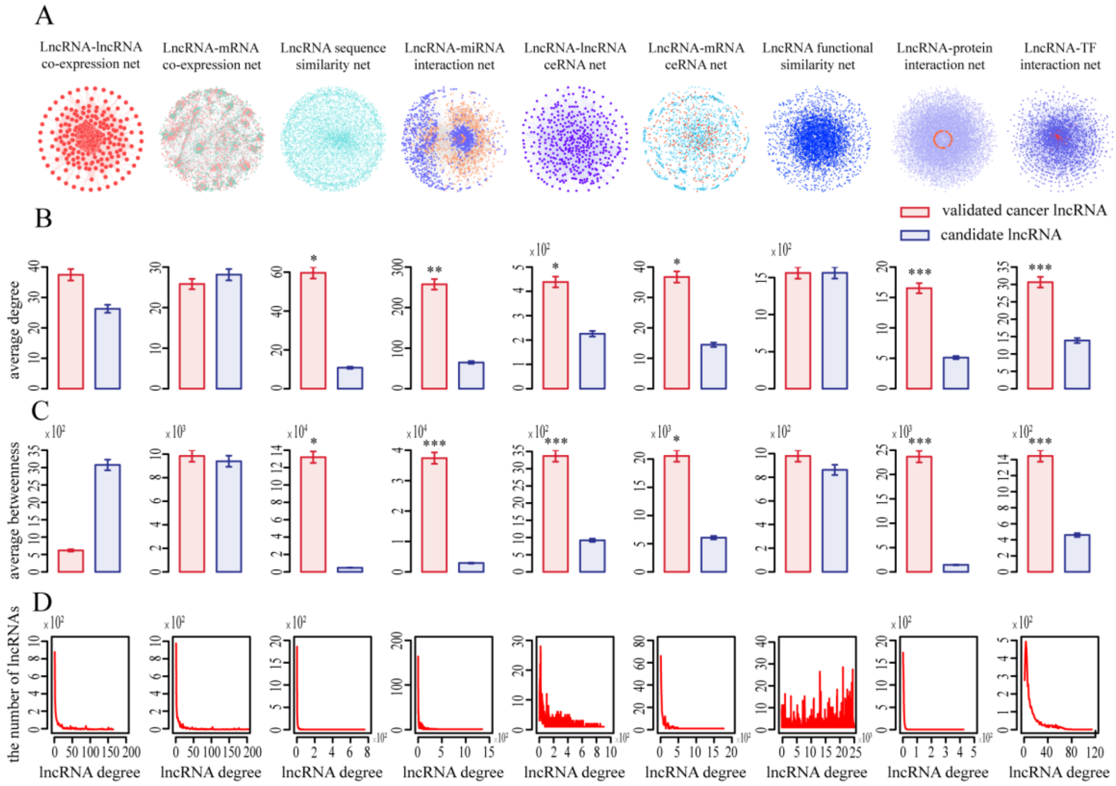

Supplementary Figure 3

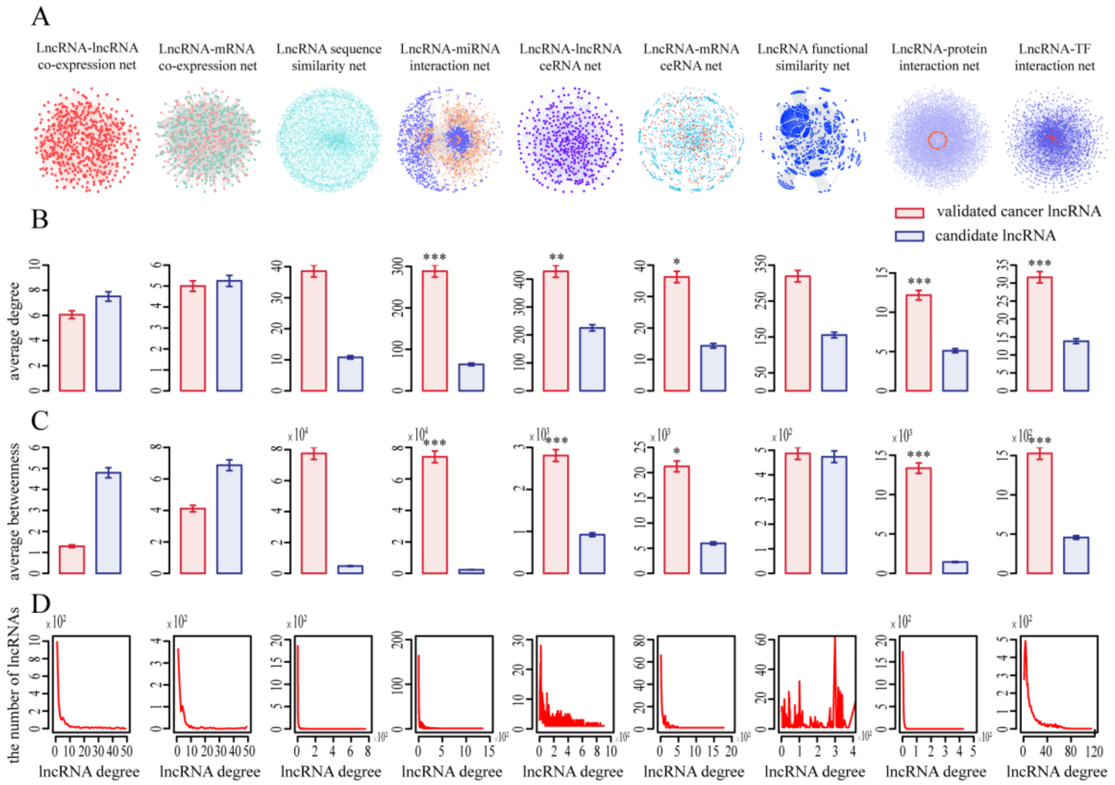

Supplementary Figure S4

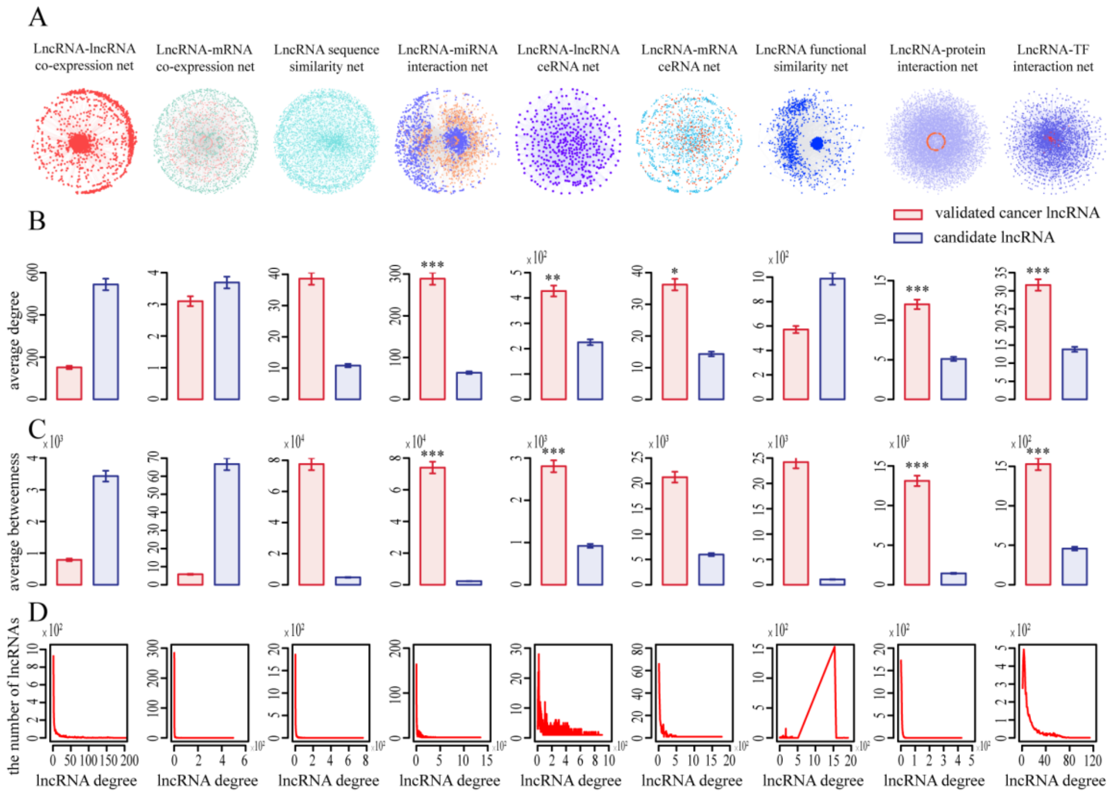

Supplementary Figure S5

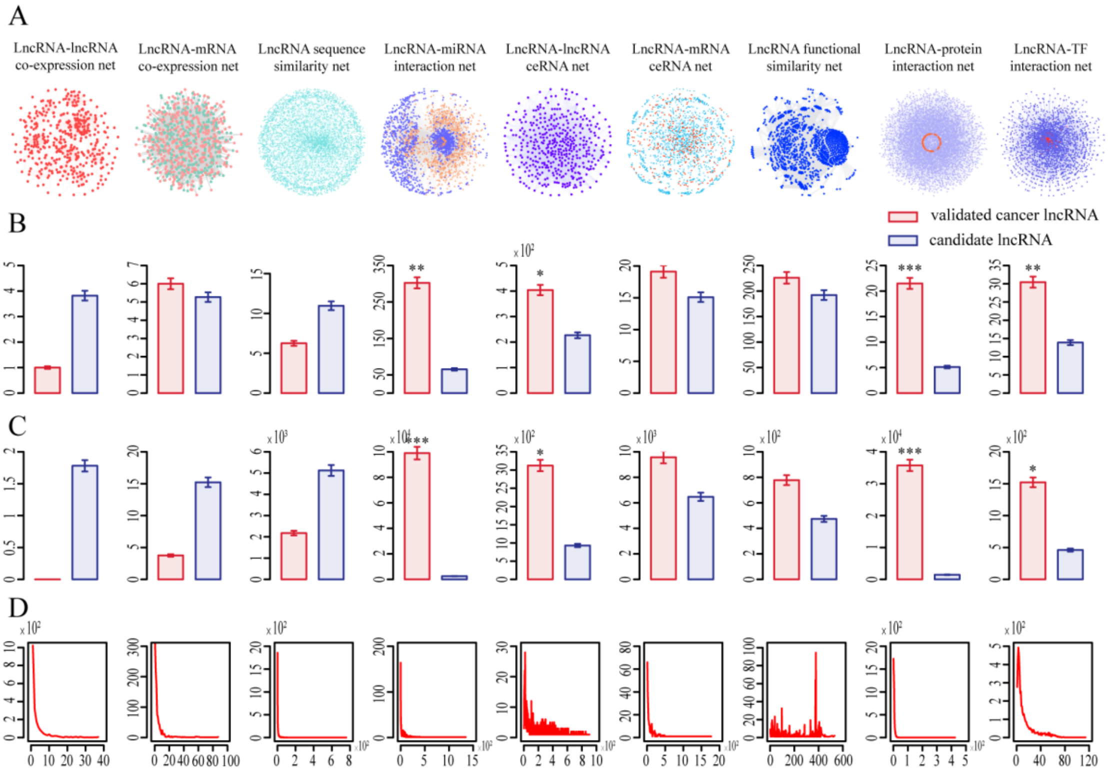

Supplementary Figure S6

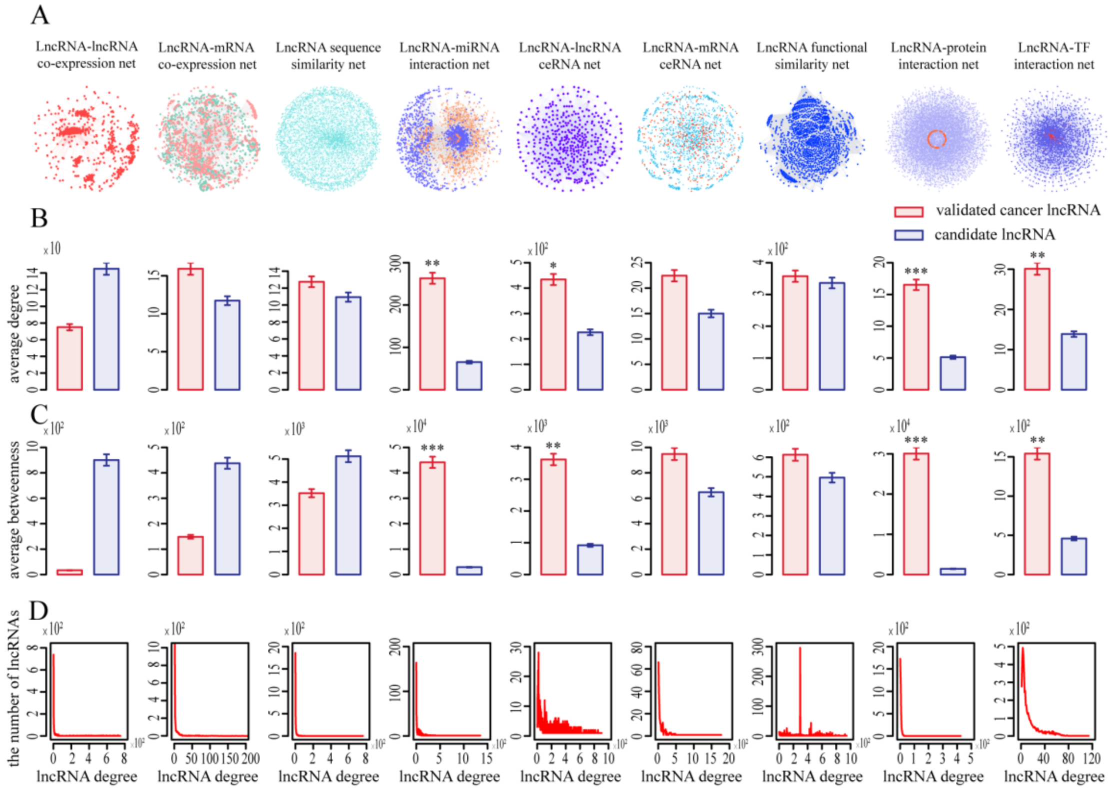

Supplementary Figure S7

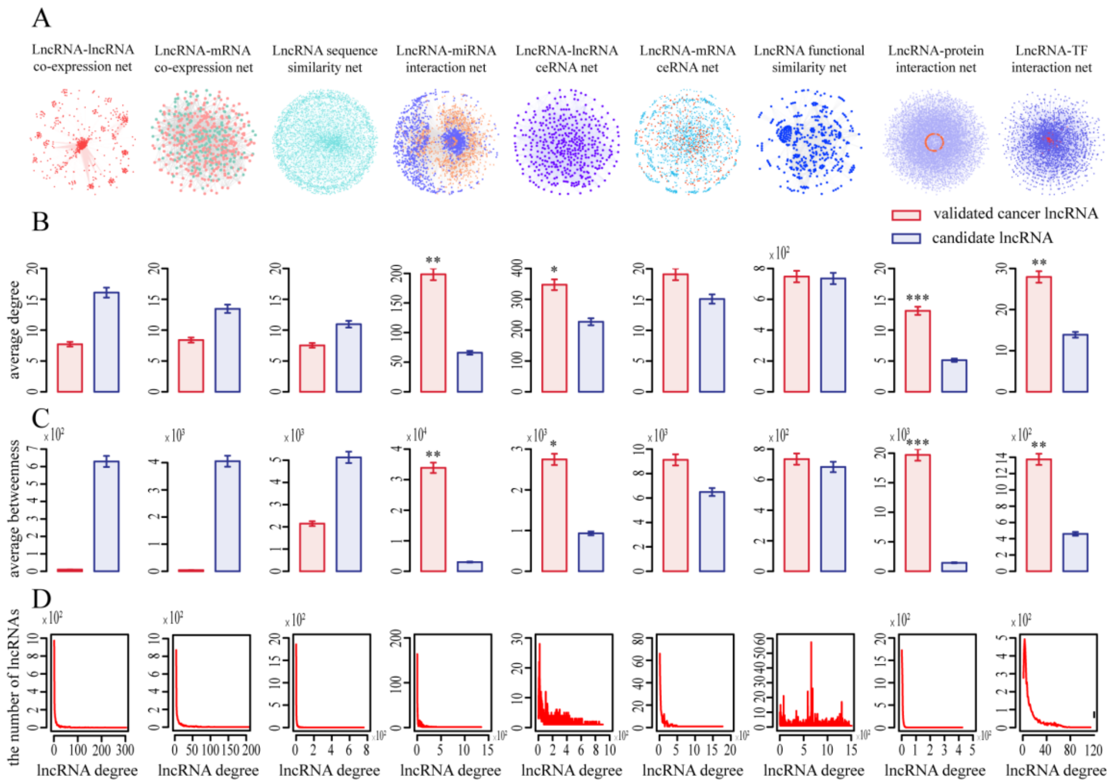

Supplementary Figure S8

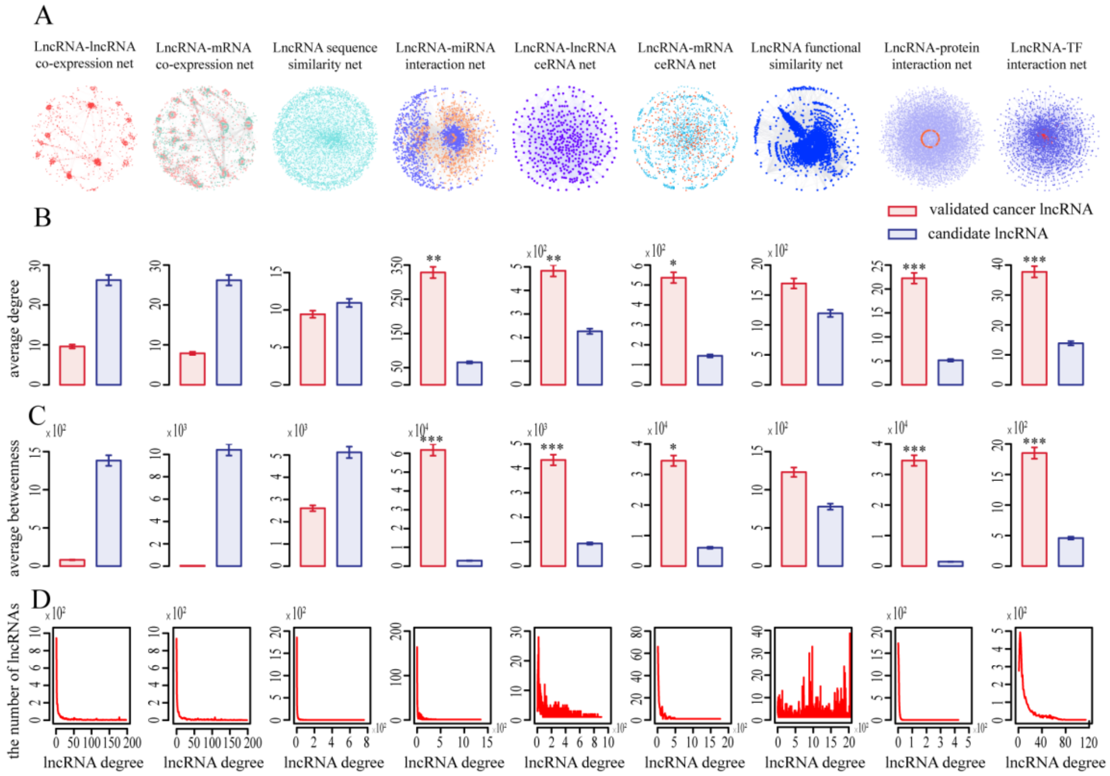

Supplementary Figure S9

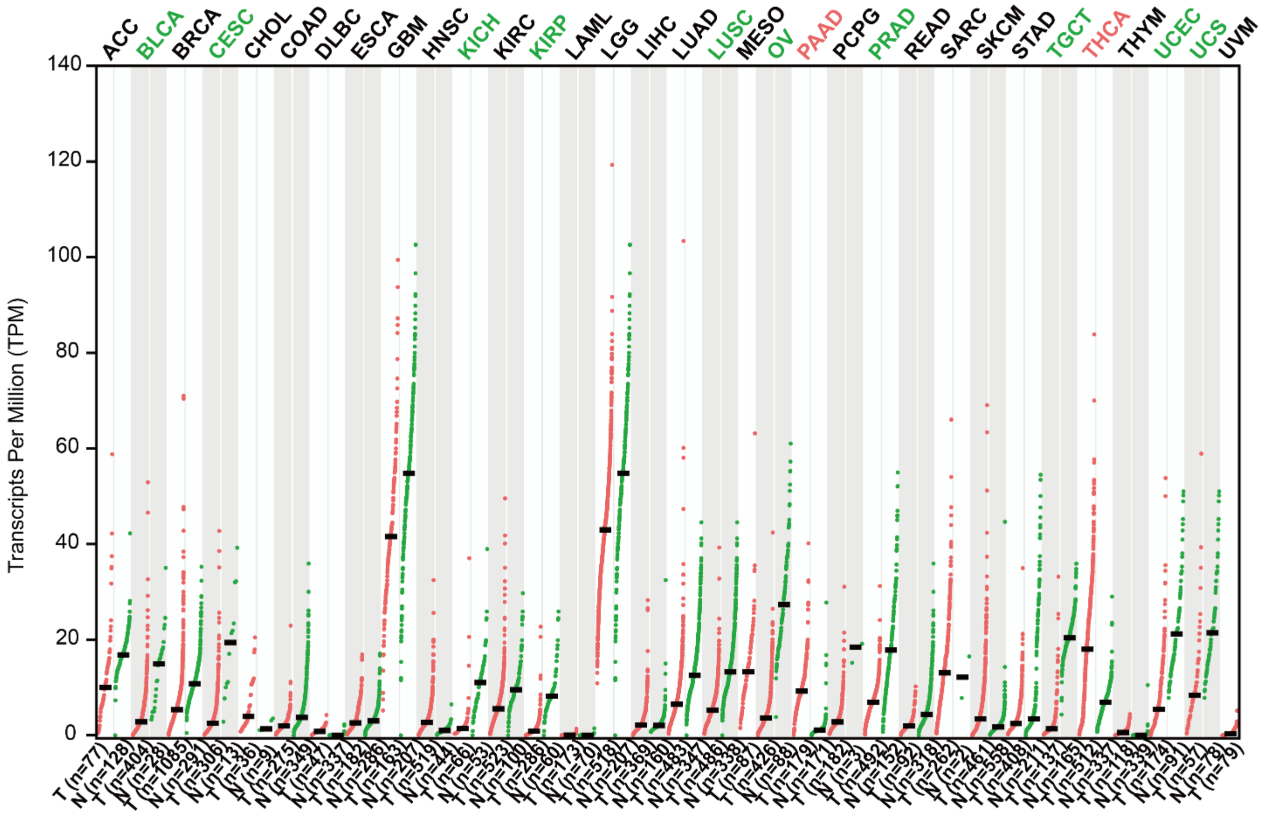

Supplementary Figure S10

Supplementary Tables

Supplementary Table S1. Number of nodes (lncRNAs) and edges involved in the nine networks across 10 cancers.

Cancer	LncRNA-lncRNA co-expression net		LncRNA-mRNA co-expression net		LncRNA sequence similarity net		LncRNA-miRNA interaction net		LncRNA-lncRNA ceRNA net		LncRNA-mRNA ceRNA net		LncRNA functional similarity net		LncRNA-protein interaction net		LncRNA-TF interaction net	
	**edge**	**node**	**edge**	**node**	**edge**	**node**	**edge**	**node**	**edge**	**node**	**edge**	**node**	**edge**	**node**	**edge**	**node**	**edge**	**node**
**BLCA**	43867	3349	84634	3237	28622	5231	109542	1634	186306	1633	5002	329	1584543	2656	53266	10355	68676	4937
**BRCA**	20595	2465	2068	596	28622	5231	109542	1634	186306	1633	5002	329	5360	486	53266	10355	68676	4937
**COAD**	153099	839	104838	1749	28622	5231	109542	1634	186306	1633	5002	329	812450	1651	53266	10355	68676	4937
**HNSC**	22808	2246	11465	798	28622	5231	109542	1634	186306	1633	5002	329	33856	700	53266	10355	68676	4937
**LIHC**	51127	3888	106257	3781	28622	5231	109542	1634	186306	1633	5002	329	2511269	3212	53266	10355	68676	4937
**LUAD**	9134	2436	5274	1005	28622	5231	109542	1634	186306	1633	5002	329	67340	866	53266	10355	68676	4937
**LUSC**	1311872	4840	9879	2679	28622	5231	109542	1634	186306	1633	5002	329	1268028	2574	53266	10355	68676	4937
**OV**	3937	2064	5994	1138	28622	5231	109542	1634	186306	1633	5002	329	97369	1013	53266	10355	68676	4937
**PRAD**	214609	2961	25865	2200	28622	5231	109542	1634	186306	1633	5002	329	286361	1704	53266	10355	68676	4937
**STAD**	23669	2947	34633	2580	28622	5231	109542	1634	186306	1633	5002	329	730946	1989	53266	10355	68676	4937

Note:

BLCA: Bladder urothelial carcinoma, BRCA: Breast invasive carcinoma, COAD: Colon adenocarcinoma, HNSC: Head and neck squamous cell carcinoma, LIHC: Liver hepatocellular carcinoma, LUAD: Lung adenocarcinoma, LUSC: Lung squamous cell carcinoma, OV: Ovarian serous cystadenocarcinoma, PRAD: Prostate adenocarcinoma, STAD: Stomach adenocarcinoma.

Supplementary Table S2. Summary of literature-verified lncRNAs.

lncRNA	Cancer	Rank by CLING	Ref.
NEAT1	BLCA	1	[1]
JPX	BRCA	4	[2]
TUG1	COAD	2	[3]
CDKN2B-AS1	COAD	7	[4]
NEAT1	LIHC	5	[5]
NEAT1	OV	2	[6]
TUG1	PRAD	4	[7]
TUG1	STAD	4	[8]
NEAT1	STAD	7	[9]
SNHG5	STAD	10	[10]

Supplementary Table S3. Time requirements of all three data integration methods.

Method	Calculating time (s)		
	1000 lncRNAs	5000 lncRNAs	10000 lncRNAs
**Endeavour**	10.18	60.88	209.26
**DRS**	2.54	8.74	40.01
**CLING**	2.03	4.76	9.17

Supplementary Table S4. CLING-ranked list of the top 20 candidate LIHC-related lncRNAs

	Ensembl ID	lncRNA name	Rank by CLING	Fold Change	P_value of DEA
*	ENSG00000228223	HCG11	1	1.0133	9.05556E-01
	ENSG00000231607	DLEU2	2	2.91372	1.62546E-15
	ENSG00000256546	AC156455	3	4.82235	2.50761E-02
	ENSG00000186019	AC084219	4	7.88079	8.27236E-05
*	ENSG00000245532	NEAT1	5	1.87706	5.57064E-09
*	ENSG00000203875	SNHG5	6	1.63019	2.82935E-05
*	ENSG00000225470	JPX	7	1.53206	8.46220E-13
	ENSG00000245910	SNHG6	8	3.67549	1.56098E-16
	ENSG00000231074	HCG18	9	2.70257	5.02553E-27
*	ENSG00000247556	OIP5-AS1	10	1.30156	1.49134E-06
*	ENSG00000229807	XIST	11	1.89379	4.86458E-03
*	ENSG00000245937	CTC-228N24	12	1.57514	3.31332E-13
*	ENSG00000179743	RP11-169K16	13	1.81327	3.27600E-13
	ENSG00000251136	RP11-37B2	14	2.88419	4.88060E-18
	ENSG00000270141	TERC	15	4.07585	2.93156E-04
	ENSG00000197989	SNHG12	16	3.57383	9.05450E-19
*	ENSG00000246430	LINC00968	17	1.52574	4.45396E-01
	ENSG00000232512	7SK	18	7.20728	4.57999E-02
*	ENSG00000186594	MIR22HG	19	0.94995	5.37193E-01
	ENSG00000225783	MIAT	20	3.34346	3.98399E-02

Note: * signifies lncRNAs that could not be identified by DEA

REFERENCES

1. XianGuo C, ZongYao H, Jun Z et al. Promoting progression and clinicopathological significance of NEAT1 over-expression in bladder cancer, Oncotarget 2016.
2. Huang YS, Chang CC, Lee SS et al. Xist reduction in breast cancer upregulates AKT phosphorylation via HDAC3-mediated repression of PHLPP1 expression, Oncotarget 2016.
3. Zhao Y, Li H, Fang S et al. NONCODE 2016: an informative and valuable data source of long non-coding RNAs, Nucleic Acids Res 2016;44:D203-208.
4. Naemura M, Tsunoda T, Inoue Y et al. ANRIL regulates the proliferation of human colorectal cancer cells in both two- and three-dimensional culture, Mol Cell Biochem 2016;412:141-146.
5. Guo S, Chen W, Luo Y et al. Clinical implication of long non-coding RNA NEAT1 expression in hepatocellular carcinoma patients, Int J Clin Exp Pathol 2015;8:5395-5402.
6. Chai Y, Liu J, Zhang Z et al. HuR-regulated lncRNA NEAT1 stability in tumorigenesis and progression of ovarian cancer, Cancer Med 2016;5:1588-1598.
7. Du Z, Sun T, Hacisuleyman E et al. Integrative analyses reveal a long noncoding RNA-mediated sponge regulatory network in prostate cancer, Nat Commun 2016;7:10982.
8. Zhang E, He X, Yin D et al. Increased expression of long noncoding RNA TUG1 predicts a poor prognosis of gastric cancer and regulates cell proliferation by epigenetically silencing of p57, Cell Death Dis 2016;7:e2109.
9. Ma Y, Liu L, Yan F et al. Enhanced expression of long non-coding RNA NEAT1 is associated with the progression of gastric adenocarcinomas, World J Surg Oncol 2016;14:41.
10. Zhao L, Guo H, Zhou B et al. Long non-coding RNA SNHG5 suppresses gastric cancer progression by trapping MTA2 in the cytosol, Oncogene 2016.
